# Botulinum Toxin A Injection for Autonomic Dysreflexia—Detrusor Injection or Urethral Sphincter Injection?

**DOI:** 10.3390/toxins15020108

**Published:** 2023-01-26

**Authors:** Po-Ming Chow, Hann-Chorng Kuo

**Affiliations:** 1 Department of Urology, National Taiwan University Hospital and College of Medicine, No. 7, Chung-Shan South Road, Taipei 100225, Taiwan; 2 Glickman Urologic and Kidney Institute, Cleveland Clinic, 9500 Euclid Avenue, Cleveland, OH 44195, USA; 3 Department of Urology, Hualien Tzu Chi Hospital, Buddhist Tzu Chi Medical Foundation and Tzu Chi University, 707, Sec. 3, Chung-Yang Rd., Hualien 970, Taiwan

**Keywords:** spinal cord injury, neurogenic bladder, onabotulinumtoxinA

## Abstract

Spinal cord injuries (SCI) have a profound impact on autonomic systems, sometimes resulting in multi-organ dysfunction, including of the neurogenic bladder. Autonomic dysreflexia (AD) is commonly seen in patients with SCI above T6 when the injured cord develops a deregulated sympathetic reflex, which can be induced by bladder sensation and can cause hypertensive crisis. While intravesical injection of botulinum toxin A (Botox) is a standard therapy for neurogenic detrusor overactivity, the role of Botox for AD has rarely been described. This study reviewed the medical records of SCI patients who reported AD and received either detrusor or urethral sphincter injection with Botox. The primary endpoint is the subjective improvement of AD. The secondary endpoint is a change in videourodynamic parameters before and after Botox injection. A total of 200 patients were enrolled for analysis. There were 125 (62.5%) patients in the detrusor injection group, and 75 (37.5%) in the urethral sphincter injection group. There were 79 (63.2%) patients in the detrusor injection group and 43 (57.3%) in the urethral sphincter injection group reporting moderate or marked improvement. Detrusor injection leads to a greater improvement in AD, probably because of decreased detrusor pressure and increased compliance after Botox injection. Urethral sphincter injection appears to have a modest effect on AD, despite general improvements in the voiding parameters of videourodynamic study.

## 1. Introduction

The spinal cord can be affected by various disorders, which may be classified into traumatic or non-traumatic. Traumatic injuries are often marked by distinct events, whereas non-traumatic injuries are caused by medical conditions, including degenerative, autoimmune, vascular, infectious, or neoplastic diseases. The leading etiology for non-traumatic disease is cervical spondylosis, followed by multiple sclerosis and tumors [1]. The most common causes of traumatic injuries are vehicular accidents and falls. According to the National Spinal Cord Injury Statistical Center (Birmingham, AL, USA), the annual incidence of traumatic spinal cord injury (SCI) is approximately 54 cases per one million people in the United States [2]. The average age is 43, and 78% of the affected patients are male. The cervical spine is the most common site of injury, comprising more than 50% of the cases [3]. 

The high prevalence of C-spine SCI results in multi-organ dysfunction. Cardiac risks are elevated due to a more prevalent adverse lipid profile, insulin resistance, and abnormal glucose metabolism in SCI patients. Pneumonia is frequent due to an impairment of the respiratory muscles and poor clearance of lung secretions. Constipation is common in patients who have injuries above the conus medullaris, resulting in hypertonic pelvic muscles. Pressure ulcers are directly related to immobility, and are difficult to manage in SCI patients [4]. The immobility resulting from either tetraplegia or paraplegia further aggravates cardiac, respiratory, metabolic, wound, and urinary complications through deprivation of exercises, muscle power reduction, sensation impairment, and fluid and nutritional imbalance. Higher mortality from the above conditions is observed in these patients due to their atypical presentations and delayed diagnosis. Multi-organ dysfunction also shortens the life expectancy of SCI patients to approximately 90 percent that of the normal population [5]. 

Bladder function is altered in SCI patients regardless of the level of the lesion [6]. In higher-level injuries, uninhibited contraction of the detrusor muscle results in detrusor hyperreflexia, with or without bladder sensation, leading to urinary incontinence, poor bladder compliance, and vesicoureteral reflux. These disorders can be worsened by the un-relaxation or dyssynergic contraction of the external sphincter during the voiding phase, which further increases intravesical pressure. In lower-level injuries, acontractile detrusors result in urinary retention, and insufficient sphincters result in urinary incontinence [7]. Both storage and voiding function require assistance to maintain a low-pressure, compliant, contractile bladder, as well as a continent sphincter. The wellness of the bladder directly reflects the quality of life in terms of the reduction in infection, stone formation, ureteral reflux, and renal function impairment [8].

Autonomic dysreflexia (AD) is a distinct cardiovascular complication commonly seen in SCI above T6. The injury separates the sympathetic neurons from the supraspinal regulation, resulting in a decentralized cord. An episode of AD presents with hypertension and concomitant baroreflex-mediated bradycardia, initiated by unmodulated sympathetic reflexes [9]. The reflex is often triggered by a stimulation below the injury level, such as constipation, bladder distention, pressure sores, or even tight clothing in SCI patients. During an episode of AD, the systolic pressure can reach as high as 325 mmHg [10]. Hypertensive crisis can result in cardiac arrest, seizure, stroke, or sudden death. In patients with SCI and neurogenic bladder, episodes of AD can be discerned by their symptoms, including headache, sweating, and flushing above the injured level.

Intravesical injection of botulinum toxin A (Botox) is a standard therapy for neurogenic detrusor overactivity (NDO). The toxin works on the neuro-muscular junction and relaxes the detrusor muscle, thus improving bladder compliance and reducing urinary incontinence [11]. Another application of Botox is urethral sphincter injection for the purpose of lowering bladder outlet resistance [12]. Both detrusor and urethral sphincter injections of Botox can theoretically improve AD by reducing intravesical pressure during storage and reducing bladder outlet resistance during voiding. However, few studies have investigated the clinical effect of Botox on AD. Schurch et al. first reported the disappearance of AD in 3 of 31 SCI patients who received botulinum-A toxin injections [13]. Fougere et al. found that AD was reduced and blood pressure was stabilized after botulinum-A toxin injections in 17 patients [14]. Herein, we report our experience with Botox injection in either the detrusor or the urethral sphincter, and its effect on AD in patients with SCI. 

## 2. Results

A total of 200 patients were enrolled for analysis. There were 125 (62.5%) patients in the detrusor injection group, and 75 (37.5%) in the urethral sphincter injection group. The average age was 40.8 years, and 131 (65.5%) were men. The levels of injuries were 52 (26%) at the C-spine and 148 (74%) at the T-spine. Symptoms included 2 (1%) with normal voiding, 71 (35.5%) with difficult voiding, 107 (53.5%) with urgency incontinence, and 32 (16%) with urinary retention. Types of bladder management among the participants included 38 (19%) self-voiding, 45 (22.5%) using diapers, 20 (10%) using abdominal pressure, 102 (51%) using percussion voiding, 41 (20.5%) using reflex voiding, 31 (15.5%) using intermittent catheterization, 11 (5.5%) using indwelling catheters, and 7 (3.5%) using cystostomy. The baseline characteristics of each group are summarized in Table 1.

The patient-reported improvements in AD are presented as GRA. Of the 200 patients, 28 (14%) reported no improvement, 50 (25%) reported mild improvement, 75 (32.5%) reported moderate improvement, and 47 (23.5%) reported marked improvement. There were more patients in the detrusor group reporting moderate or marked improvement in AD (Table 2). AD was found to increase immediately after intradetrusor injection in two patients, but in none after urethral sphincter injection. However, there were no patients who reported having a worsened GRA of AD after treatment at the follow-up time-point in this study.

At baseline, the patients in the detrusor groups had more sensitive bladders, evidenced by a smaller filling volume at first sensation (171 mL vs. 210 mL, *p* = 0.019), urge sensation (189 mL vs. 235 mL, *p* = 0.015), as well as maximal bladder capacity (254 mL vs. 293 mL, *p* = 0.038). After Botox injection, the urethral group showed significant increases in Qmax, voided volume, and VE (Table 3).

In the detrusor group, patients who had moderate or marked improvement in AD had poorer bladder compliance (31.14 vs. 70.47, *p* < 0.001), higher Pdet (45.42 vs. 23.48 cmH2O, *p* < 0.001), and higher BOOI (35.93 vs. 9.83, *p* <0.001) at baseline compared to patients who had no or mild improvement. The post-treatment changes of the moderate or marked improvement subgroups and those of the no or mild subgroups were significantly different in bladder compliance and BOOI (Table 4). DO was present in 77 (61.6%) patients before Botox treatment and in 69 (55.2%) patients after Botox treatment (*p* = 0.052). DSD was present in 61 (48.8%) patients before Botox treatment and in 53 (42.4%) patients after Botox treatment (*p* = 0.268). 

In the urethral sphincter group, patients who had moderate or marked improvement in AD had marginally higher Pdet (41.65 vs. 29.13 cmH2O, *p* = 0.050) and lower capacity (258.86 vs. 399.41 ml, *p* = 0.023) compared to patients who had no or mild improvement (Table 5). DO was present in 38 (50.7%) patients before Botox treatment and in 33 (44%) patients after Botox treatment (*p* = 0.180). DSD was present in 30 (40%) patients before Botox treatment and in 20 (26.7%) patients after Botox treatment (*p* = 0.031).

## 3. Discussion

Our study revealed that Botox injection to either the detrusor or the urethral sphincter achieved moderate or marked improvement in AD in 61% of SCI patients. There were more patients with marked improvement in the detrusor group, indicating a better control of AD. The baseline VUDS profile suggested that patients with poorer bladder compliance and higher detrusor pressure showed better responses to detrusor injection, and this response was best reflected by an increase in post-treatment compliance and a decrease in DO. The benefits that these patients might report could be related to their inferior pre-treatment conditions, as these treatments did not help patients with borderline bladder dysfunction. In the urethral sphincter group, there were general improvements in VUDS parameters, including Qmax, VE, and BOOI, regardless of the subjective improvements in AD after Botox injection. To our knowledge, this is the first study that has correlated VUDS findings and AD symptom improvements in SCI patients who received Botox injection at different sites.

The leading cause of death in SCI patients has shifted from urinary complications to cardiovascular events [15], marking the importance of the management of AD. Since AD is highlighted by episodic hypertension, treatments for AD has been focused on blood pressure control with nitrites [16], calcium channel blockers [17], and alpha-adrenergic blockers [18]. However, the pathophysiology of AD includes a serial remodeling of the autonomic system: loss of supraspinal control over the sympathetic preganglionic neurons [19], synaptic reorganization of the sympathetic preganglionic neurons [20], primary afferent sprouting [21], and propriospinal plasticity [22]. This sensitized bladder proprioception, as well as other stimuli below the level of injury, are amplified to form an unregulated sympathetic reflex, resulting in an episode of AD. Considering the pathophysiology of AD, blood pressure control alone does not provide to-the-target management.

Botox paralyzes smooth or striated muscles through its inhibition of acetylcholine release in neuromuscular junctions. Through this mechanism of action, Botox has demonstrated effectiveness in reducing DO and urethral sphincter spasticity [23]. In addition to motor inhibition, Botox also has effects on the sensory neurons. The application of a sensory blockade has been proven effective in patients with bladder pain syndrome treated with intravesical injections [24]. These mechanisms include a decrease in both the release of neurotransmitters and the expression of nociceptors, as well as the suppression of afferent nerve sprouting and reorganization [25]. The diverse mechanisms of action make Botox an ideal therapy for SCI patients, as it targets both lower urinary tract symptoms and AD.

In our cohort of SCI patients who had AD that required anti-hypertensive management, LUTS-directed Botox injection yielded a 61% moderate or marked improvement in AD symptoms. There were two prior series addressing the role of Botox on AD. In the study by Schurch et al. evaluating the effect of Botox on LUTS, AD associated with bladder emptying that manifested as a hypertensive crisis during voiding disappeared after treatment in the three patients with tetraplegia [13]. Although this was a prospective study, AD was not an end point, but an incidental finding. Another study by Fougere et al. prospectively measured blood pressure during UDS and daily activity. The authors found that the amplitude of UDS-induced hypertension was attenuated in 17 patients after Botox injection; however, there were no significant differences found in 24-h ambulatory blood pressure monitoring [14]. 

Our results suggest that improvement in AD can be more significant in those who have poorer bladder compliance and higher Pdet at baseline, which are typical UDS indications for Botox injection. This finding implies that additional detrusor injection can be considered in patients symptomatically indicated for urethral injection, in order to further eliminate their AD symptoms. Nonetheless, there were still 14% of patients who reported no improvement in AD, indicating insufficient management for either LUTS or other stimulatory conditions such as constipation or pressure sores.

The strengths of our study are the large number of cases and complete VUDS evaluations. There are some limitations to our study. First, the baseline AD severity was unclear, and was not objectively measured. As there are currently no symptom scores or other objective evaluation tools for AD, we relied on patient-reported general assessments of the outcome to evaluate the treatment responses. Some studies used ambulatory blood pressure monitoring, but this method may not always record the blood pressure during AD episodes. Furthermore, there is no consensus on the criteria for blood pressure elevation. Second, this was not a randomized trial, and the decision for detrusor or urethral sphincter injection was based on the patients’ main lower urinary tract symptoms and requirements. Although there has been no data suggesting predisposing factors for AD, significant bias might result from any unbalanced factors, such as age, sex, or the injury level between the two arms.

## 4. Conclusions

Detrusor or urethral sphincter injection of Botox were both shown to improve AD in the majority of SCI patients. Detrusor injection leads to a greater improvement in AD, probably because of decreased detrusor pressure and increased bladder compliance. Urethral sphincter injection appears to have a modest effect on AD, despite general improvement in the VUDS parameters.

## 5. Methods

### 5.1. Ethical Approval

The study was approved by the Institutional Review Board of Hualien Tzu Chi Hospital (IRB 110-033-B). Patients’ informed consent was waived due to the retrospective nature of this study.

### 5.2. Patient Enrollment

We retrospectively reviewed the medical records of SCI patients who reported AD and received either detrusor or urethral sphincter injection with Botox from 1998 to 2022. All patients had either storage symptoms, such as urgency and urgency incontinence; emptying symptoms, such as difficult urination or large postvoid residual volume; or both storage and emptying symptoms. Thus, all were ready for detrusor or urethral Botox injection. All patients also had symptoms of AD, such as headache, hypertension, increased reflexes, profuse sweating, bradycardia, and other systemic symptoms either associated with bladder fullness or occurring during urination. These AD symptoms were considered moderate to severe, causing discomfort to the patients and requiring medication to alleviate them. Patient data included age, sex, level of SCI, bladder management, videourodynamic study (VUDS) profiles, and subjective improvement in AD.

### 5.3. Botulinum Toxin A Injection

The techniques for Botox injection were described previously [12,26,27]. The treatment was performed in an operating room under light intravenous general anesthesia. For detrusor injection, 200 U of onabotulinumtoxinA (Botox, Allergan, Irvine, CA, USA) was diluted with 20 mL of normal saline and injected into 20 well-distributed sites in the bladder wall, sparing the trigone. All cystoscopic injections were performed using a rigid injection instrument (22-Fr, Richard Wolf, Knittlingen, Germany) and a 23-gauge injection needle. For urethral sphincter injection, a total of 100 U Botox was given. A single vial of 100 U Botox was dissolved in 5 mL of normal saline, resulting in a concentration equivalent to 20 U/mL. Each 1 mL of Botox solution was injected transurethrally under cystoscopy into the urethral sphincter at the 2, 4, 8, 10, and 12 o’clock positions in men, and transcutaneously into the urethral sphincter along the urethral lumen at the sides of the urethral meatus in women. The selection of Botox injection to the detrusor or the urethral sphincter was based on the individual patient’s main lower urinary tract symptoms. Detrusor Botox injection was performed for NDO with urinary incontinence and AD. Urethral sphincter Botox injection was performed for detrusor sphincter dyssynergia (DSD) to facilitate spontaneous voiding, ease self-catheterization, and improve AD.

### 5.4. VUDS Parameters

VUDS was performed under fluoroscopy and pressure flow study [28]. The VUDS parameters were defined as follows. Bladder sensation was evaluated by first sensation of filling (FSF), full sensation (FS), and urge sensation (US). Cystometric bladder capacity (CBC) was calculated by adding post-voiding residual (PVR) and voided volume (Vol). Bladder compliance was calculated by dividing the volume at full sensation (FS) by the detrusor pressure (Pdet). Voiding efficiency (VE) was defined as voided volume divided by bladder capacity. Maximal flow rate (Qmax) and detrusor pressure at the maximum flow rate (Pdet@Qmax) were recorded. The bladder outlet obstruction index (BOOI) was calculated by (Pdet@Qmax -2 × Qmax). Detrusor overactivity (DO) was defined as any involuntary detrusor contraction during the filling phase of the pressure flow study [29].

### 5.5. Outcome Measurement and Statistics Analysis

The primary endpoint was the subjective improvement in AD, as defined by the global response assessment (GRA). The scale we used was as follows: −3 for markedly worse, −2 for moderately worse, −1 for mildly worse, 0 for no change, 1 for mildlimprovement, 2 for moderate improvement, and 3 for marked improvement [30]. The GRA assessments were taken within 1 month after Botox, and VUDS was carried out within 3 months after Botox. GRA and VUDS were evaluated separately. The secondary endpoint was the change in VUDS parameters before and after Botox injection. Either the Student’s *t*-test or Mann–Whitney U test was performed to compare numerical data, and the Chi-square test was performed to compare categorical data. Statistical analyses were performed using free software (R version 4.0.0). All statistical tests were two-tailed, with *p* < 0.05 indicating significance.

## Figures and Tables

**Table 1:** Baseline characteristics in detrusor injection and urethral sphincter injection groups.

	Detrusor (*n* = 125)	Sphincter (*n* = 75)	*p*-Value *
Age	37.75 (12.67)	45.90 (15.78)	**<0.001**
Sex			0.071
Men	76 (60.8%)	55 (73.3%)	
Women	49 (39.2%)	20 (26.7%)	
SCI level			0.067
Cervical	27 (21.6%)	25 (33.3%)	
Thoracic	98 (78.4%)	50 (66.7%)	
Symptom			
Normal voiding	2 (1.6%)	0 (0.0%)	0.529
Difficult voiding	23 (18.4%)	48 (64.0%)	**<0.001**
Urge incontinence	92 (73.6%)	15 (20.0%)	**<0.001**
Retention	17 (13.6%)	15 (20.0%)	0.232
Bladder management			
Spontaneous voiding	28 (22.4%)	10 (13.3%)	0.114
On diaper	27 (21.6%)	18 (24.0%)	0.694
Abdominal pressure	10 (8.0)	10 (13.3%)	0.224
Percussion voiding	63 (50.4%)	39 (52.0%)	0.827
Reflex voiding	29 (23.2%)	12 (16.0%)	0.222
CIC/CISC	21 (16.8%)	10 (13.3%)	0.512
Urethral Foley	6 (4.8%)	5 (6.7%)	0.575
Cystostomy	2 (1.6%)	5 (6.7%)	0.105

* *p*-value by Student’s *t* test and Chi-square test.

**Table 2:** Subjective improvement after treatment.

	Detrusor (*n* = 125)	Sphincter (*n* = 75)	*p*-Value *
Satisfaction with treatment			**0.019**
No improvement	20 (16.0%)	8 (10.7%)	
Mild improvement	26 (20.8%)	24 (32.0%)	
Moderate improvement	42 (33.6%)	33 (44.0%)	
Marked improvement	37 (29.6%)	10 (13.3%)	

*p-value by Chi-square test.

**Table 3:** Baseline and post-treatment videourodynamic parameters in the detrusor and urethral Botox injection groups.

VUDS Parameters		Detrusor (*n* = 125)	Urethra (*n* = 75)	*p*-Value *
**PVR**	Baseline Post-BTX Change	184.75 (173.29) 279.77 (195.26) 97.27 (247.97)	210.16 (156.01) 223.41 (227.62) 26.82 (271.63)	0.106 0.032 0.033
**FSF**	Baseline Post-BTX Change	131.29 (86.16) 152.17 (92.55) 26.87 (103.29)	152.08 (97.10) 155.11 (97.28) 14.86 (114.71)	0.116 0.854 0.534
**FS**	Baseline Post-BTX Change	171.71 (106.12) 207.53 (115.40) 41.12 (135.68)	210.79 (121.92) 215.95 (129.10) 27.18 (147.79)	0.019 0.904 0.557
**US**	Baseline Post-BTX Change	189.32 (120.83) 226.30 (127.85) 45.72 (148.35)	235.41 (137.31) 241.64 (134.91) 32.64 (158.09)	0.015 0.540 0.699
**Compliance**	Baseline Post-BTX Change	45.61 (59.52) 38.22 (44.80) −12.12 (76.48)	55.47 (69.86) 44.14 (81.95) −12.28 (108.99)	0.106 0.857 0.434
**Pdet**	Baseline Post-BTX Change	37.34 (23.32) 26.06 (18.63) −10.60 (24.02)	36.31 (24.54) 27.25 (20.25) −11.7 (26.67)	0.735 0.838 0.943
**Qmax**	Baseline Post-BTX Change	5.51 (5.97) 4.10 (5.60) −1.73 (6.21)	4.69 (5.17) 6.55 (9.44) 2.25 (7.88)	0.329 0.128 0.017
**Vol**	Baseline Post-BTX Change	67.98 (81.50) 61.47 (93.53) −8.41 (103.13)	83.07 (113.29) 112.23 (137.08) 44.86 (152.88)	0.949 0.050 0.029
**CBC**	Baseline Post-BTX Change	254.11 (164.37) 341.23 (177.62) 88.86 (217.34)	293.23 (153.51) 335.64 (188.11) 71.68 (257.48)	0.038 0.652 0.290
**VE**	Baseline Post-BTX Change	0.33 (0.33) 0.21 (0.30) -0.11 (0.37)	0.29 (0.31) 0.38 (0.40) 0.12 (0.42)	0.354 0.027 0.004
**BCI**	Baseline Post-BTX Change	64.89 (39.83) 46.58 (36.85) −19.27 (46.25)	59.77 (36.44) 59.98 (51.83) −0.45 (48.25)	0.411 0.121 0.044
**BOOI**	Baseline Post-BTX Change	26.32 (25.00) 17.85 (19.56) −7.14 (22.17)	26.92 (26.17) 14.16 (27.33) −16.20 (30.57)	0.995 0.491 0.171

BCI: bladder contractility index, BOOI: bladder outlet obstruction index, CBC: cystometric bladder capacity, FS: full sensation, FSF: first sensation of filling, Pdet: detrusor pressure at maximum flow rate, PVR: post-voiding residual, Qmax: maximal flow rate, US: urge sensation, VE: voiding efficiency, * *p*-value by Mann–Whitney U test.

**Table 4:** Baseline and post-treatment videourodynamic parameters in the detrusor Botox injection patients.

VUDS Parameters		No/Mild Improvement (*n* = 46)	Moderate/Marked Improvement (*n* = 79)	*p*-Value *
**PVR**	Baseline Post-BTX Change	184.26 (184.33) 248.55 (185.82) 53.39 (238.40)	185.04 (167.75) 299.21 (200.17) 124.58 (252.10)	0.678 0.260 0.164
**FSF**	Baseline Post-BTX Change	130.17 (86.27) 174.30 (106.29) 41.76 (122.45)	131.94 (86.63) 138.40 (80.89) 17.60 (89.35)	0.959 0.159 0.657
**FS**	Baseline Post-BTX Change	183.33 (110.70) 224.70 (125.42) 40.67 (150.21)	164.95 (103.48) 196.85 (108.57) 41.40 (127.30)	0.388 0.419 0.950
**US**	Baseline Post-BTX Change	201.72 (127.17) 243.09 (134.12) 42.64 (163.30)	182.10 (117.20) 215.85 (123.92) 47.64 (139.84)	0.414 0.429 0.972
**Compliance**	Baseline Post-BTX Change	70.47 (78.70) 27.60 (21.93) −44.04 (84.90)	31.14 (38.48) 44.83 (53.56) 7.76 (63.85)	<0.001 0.212 0.003
**Pdet**	Baseline Post-BTX Change	23.48 (14.59) 22.12 (17.12) −3.00 (20.01)	45.42 (23.72) 28.51 (19.27) −15.34 (25.24)	<0.001 0.108 0.015
**Qmax**	Baseline Post-BTX Change	6.83 (7.41) 5.73 (6.40) −0.97 (6.66)	4.74 (4.83) 3.09 (4.83) −2.21 (5.92)	0.181 0.038 0.261
**Vol**	Baseline Post-BTX Change	83.39 (93.17) 89.82 (119.91) 14.58 (110.90)	59.00 (73.00) 43.81 (67.99) −22.72 (96.29)	0.290 0.043 0.184
**CBC**	Baseline Post-BTX Change	267.65 (166.98) 338.36 (161.02) 67.97 (212.74)	246.23 (163.37) 343.02 (188.70) 101.87 (221.17)	0.467 0.821 0.520
**VE**	Baseline Post-BTX Change	0.37 (0.35) 0.28 (0.33) −0.05 (0.31)	0.31 (0.32) 0.17 (0.27) −0.15 (0.40)	0.396 0.050 0.091
**BCI**	Baseline Post-BTX Change	57.61 (44.93) 50.76 (40.83) −7.85 (46.91)	69.13 (36.17) 43.98 (34.28) −26.38 (44.82)	0.032 0.460 0.045
**BOOI**	Baseline Post-BTX Change	9.83 (16.08) 10.67 (17.81) −1.06 (17.38)	35.93 (24.30) 22.32 (19.41) −10.92 (24.07)	<0.001 0.004 0.044

BCI: bladder contractility index, BOOI: bladder outlet obstruction index, CBC: cystometric bladder capacity, FS: full sensation, FSF: first sensation of filling, Pdet: detrusor pressure at maximum flow rate, PVR: post-voiding residual, Qmax: maximal flow rate, US: urge sensation, VE: voiding efficiency. * *p*-value by Mann–Whitney U test.

**Table 5:** Baseline and post-treatment VUDS parameters in the urethral sphincter Botox injection patients.

VUDS Parameters		No/Mild Improvement (*n* = 32)	Moderate/Marked Improvement (*n* = 43)	*p*-Value *
PVR	Baseline Post-BTX Change	235.22 (169.81) 213.33 (221.92) −33.61 (262.36)	191.51 (144.09) 230.38 (235.58) 68.65 (275.05)	0.304 0.905 0.145
FSF	Baseline Post-BTX Change	170.88 (107.03) 182.17 (71.61) 16.61 (107.40)	138.09 (87.67) 136.38 (109.08) 13.65 (121.59)	0.210 0.016 0.738
FS	Baseline Post-BTX Change	228.69 (122.39) 250.28 (131.10) 35.44 (149.40)	197.47 (121.27) 192.19 (124.68) 21.46 (149.36)	0.197 0.061 0.793
US	Baseline Post-BTX Change	255.91 (139.08) 278.00 (146.92) 37.33 (158.65)	220.16 (135.60) 216.46 (122.53) 29.38 (160.76)	0.161 0.145 0.793
Compliance	Baseline Post-BTX Change	61.60 (68.64) 40.05 (61.28) −25.21 (87.26)	50.90 (71.22) 46.97 (94.76) −3.33 (122.68)	0.197 0.793 0.233
Pdet	Baseline Post-BTX Change	29.13 (19.86) 26.39 (22.32) −3.28 (27.10)	41.65 (26.48) 27.85 (19.12) −17.54 (25.24)	0.050 0.667 0.173
Qmax	Baseline Post-BTX Change	4.78 (6.08) 7.22 (11.07) 3.17 (7.64)	4.63 (4.44) 6.08 (8.33) 1.62 (8.13)	0.621 0.636 0.381
Vol	Baseline Post-BTX Change	104.19 (140.18) 137.67 (155.35) 52.06 (192.16)	67.35 (86.69) 94.62 (122.97) 39.88 (122.58)	0.768 0.436 0.624
CBC	Baseline Post-BTX Change	339.41 (165.07) 351.00 (182.20) 18.44 (274.19)	258.86 (136.29) 325.00 (194.93) 108.54 (243.83)	0.023 0.489 0.252
VE	Baseline Post-BTX Change	0.31 (0.33) 0.39 (0.41) 0.13 (0.43)	0.28 (0.29) 0.38 (0.39) 0.12 (0.41)	0.965 0.855 0.877
BCI	Baseline Post-BTX Change	53.03 (36.40) 62.50 (53.66) 12.56 (41.08)	64.79 (36.08) 58.23 (51.52) −9.46 (51.49)	0.148 0.793 0.079
BOOI	Baseline Post-BTX Change	19.56 (23.23) 11.94 (35.52) −9.61 (34.22)	32.40 (27.14) 15.69 (20.52) −20.77 (27.53)	0.056 0.821 0.283

BCI: bladder contractility index, BOOI: bladder outlet obstruction index, CBC: cystometric bladder capacity, FS: full sensation, FSF: first sensation of filling, Pdet: detrusor pressure at maximum flow rate, PVR: post-voiding residual, Qmax: maximal flow rate, US: urge sensation, VE: voiding efficiency. * *p*-value by Mann–Whitney U test.

## Data Availability

All data were presented with the manuscript.
